# Epigenetic Modifications as Biomarkers of Tumor Development, Therapy Response, and Recurrence across the Cancer Care Continuum

**DOI:** 10.3390/cancers10040101

**Published:** 2018-04-01

**Authors:** Margaret L. Thomas, Paola Marcato

**Affiliations:** 1 Department of Pathology, Dalhousie University, Halifax, NS B3H 4G7, Canada; meg.thomas@dal.ca; 2 Department of Microbiology and Immunology, Dalhousie University, Halifax, NS B3H 4G7, Canada

**Keywords:** epigenetics, biomarkers, cancer, liquid biopsy

## Abstract

Aberrant epigenetic modifications are an early event in carcinogenesis, with the epigenetic landscape continuing to change during tumor progression and metastasis—these observations suggest that specific epigenetic modifications could be used as diagnostic and prognostic biomarkers for many cancer types. DNA methylation, post-translational histone modifications, and non-coding RNAs are all dysregulated in cancer and are detectable to various degrees in liquid biopsies such as sputum, urine, stool, and blood. Here, we will focus on the application of liquid biopsies, as opposed to tissue biopsies, because of their potential as non-invasive diagnostic tools and possible use in monitoring therapy response and progression to metastatic disease. This includes a discussion of septin-9 (*SEPT9*) DNA hypermethylation for detecting colorectal cancer, which is by far the most developed epigenetic biomarker assay. Despite their potential as prognostic and diagnostic biomarkers, technical issues such as inconsistent methodology between studies, overall low yield of epigenetic material in samples, and the need for improved histone and non-coding RNA purification methods are limiting the use of epigenetic biomarkers. Once these technical limitations are overcome, epigenetic biomarkers could be used to monitor cancer development, disease progression, therapeutic response, and recurrence across the entire cancer care continuum.

## 1. Introduction

Cancer has been referred to as “cellular chaos”. This is an appropriate description for a disease which is characterized by uncontrolled cell proliferation and avoiding the host’s strategies to eliminate aberrant cells. Part of the chaotic nature of cancer cells is that though all cancers share certain hallmark traits, the driving forces and resulting phenotype of the aberrant cells can vary greatly [1]. While the role of genetic mutations as drivers of carcinogenesis has been firmly established, epigenetic modifications have more recently been proposed as important drivers of cancer [2].

The term epigenotype was first coined by C.H. Waddington in 1942 to describe the heritable alterations in gene expression which affect phenotype and do not change the DNA sequence itself [3]. Epigenetic modifications are key regulators of gene expression and also contribute to genomic stability/chromatin structure. Regardless of whether aberrant epigenetic modifications are required for carcinogenesis, certain modifications are consistently dysregulated among cancers. This presents an opportunity to use these modifications as biomarkers for screening, detection, prediction of therapeutic response, and relapse surveillance.

### 1.1. DNA Methylation

The nucleotide alphabet has been expanded beyond ATGC with the discovery of modified bases, the best-characterized of which is 5-methylcytosine (5mC) [4]. In 5mC, a methyl moiety, donated by *S*-adenosylmethionine (SAM), is added to the 5′ position of a cytosine residue in CpG dinucleotides [5]. The maintenance and de novo generation of 5mC is mediated by DNA methyltransferases DNMT1/3A/3B. Genomic regions with high concentration of CpGs are known as CpG islands and seem to have an important role in gene expression regulation. Approximately 40–60% of human genes have CpG islands in the promoter region; and when these islands acquire 5mC, transcription of the gene is inhibited. Recently, 5mC-mediated transcriptional repression was also observed in genes without promoter CpG islands [6,7,8]. A prototypical cancer phenotype consists of a globally hypomethylated genome (which disrupts genomic stability) concurrent with promoter-specific hypermethylation (which silences tumor suppressor genes) [2,9,10].

Several methods exist for assessing DNA methylation at a global or CpG site-specific level. Global methylation levels can be determined via liquid chromatography–electrospray ionization–tandem mass spectrometry, luminometric methylation assay, or using methylation of repetitive sequences like long interspersed nuclear element (LINE) as a proxy of global methylation [11,12,13]. Site-specific methylation assays are more prevalent in clinical biomarker studies and can use either genome-wide discovery/screening approaches (e.g., Illumina beadchip, reduced representation or whole genome bisulfite sequencing, or methylated DNA immunoprecipitation sequencing), or can be used to investigate a single region (e.g., pyrosequencing or methylation-specific polymerase chain reaction (MS-PCR)) [14,15]. The most commonly used assays by far are MS-PCR and the Illumina HumanMethylation bead kits [16,17]. The Illumina kits are typically used for genome-wide searches for methylation biomarkers, while a clinical assay will likely resemble MS-PCR, which assays a single CpG island. 

### 1.2. Post-Translational Histone Modifications

The human genome consists of approximately 3 billion base pairs and is able to fit within a cell due to the tightly regulated process of DNA compaction, the first stage of which is based around the nucleosome. The nucleosome is a core unit of chromatin consisting of an octamer of four histone proteins (H2A, H2B, H3, and H4) with approximately 147 bp of DNA wrapped twice around the complex [18]. An amino acid tail extends from each histone, and it is post-translational modifications to these tails which affect histone–DNA interactions and nuclear architecture [19]. Over 60 distinct histone modifications exist, though most cancer-related research focuses on acetylation (mediated by histone deacetylases (HDACs) and histone acetyltransferases (HATs)) and methylation (mediated by several protein lysine methyl-transferases like polycomb repression complex) [20]. 

The presence of these modifications forms a histone “code” that can affect transcriptional activity of the associated DNA sequence via directly impacting DNA wrapping or through recruiting enzyme complexes to wrap the DNA [21]. Histone modifications which generally indicate areas of active transcription include H3K4me (methylation of histone 3 at lysine position 4), H3K36me, H3KAc, H4K16Ac, and H3KAc; while H3K27me and H3K20me are associated with gene repression [21,22,23,24]. Cancer cells display silencing histone modifications on tumor-suppressive genes and encourage active transcription histone modifications on oncogenes [25]. Global histone profiling revealed a general loss of H4K16Ac and H4K20me3 (trimethylation) in a variety of cancers [25,26]. As we improve our understanding of the role histone modifications play in cancer biology, histone-based biomarkers could potentially be used in the cancer care continuum [27]. 

Dysregulation of histone variants has also been observed in cancer. These proteins are functionally distinct from the canonical replication-coupled core histones and endow special properties to chromatin. For example, H2A.Bbd incorporation results in nucleosomes containing 118–130 bp; this less compact chromatin is potentially more transcriptionally active [28]. Histone variants can also be post-translationally modified; thus the many combinations of canonical and variant histones (with their associated post-translational modifications) form a “variant network” to epigenetically alter chromatin structure and transcription [29]. Expression or mutation of specific histone variants are prognostic biomarkers, as in the case of H2A.Z in melanoma and breast where overexpression confers a poor prognosis [30,31], macroH2A expression which is lost during anal carcinoma progression [32]; or mutant H3., which is a common driving event in pediatric brain tumors [33,34]. 

Methods for quantifying histone modifications fall into two broad categories: methods designed to elucidate histone modifications affecting specific nucleosomes/loci and methods to determine global levels of a histone modification in a sample. Chromatin immunoprecipitation (ChIP) using antibodies specific to the histone modification of interest followed by sequencing of the DNA associated with the isolated histone modification (ChIP-Seq) is the most commonly used approach to investigate specific histone–DNA interactions [21,35]. Global levels can be determined using relatively simple Western blot or enzyme-linked immunosorbent assay (ELISA)-based methods [36].

### 1.3. Non-Coding RNAs

With the discovery that only approximately 3% of transcribed RNAs were subsequently translated into proteins, there was a surge of interest in the role of the non-coding RNA transcriptome [37]. There are several types of non-coding RNAs, such as small nucleolar RNAs (snoRNAs), and short interfering RNAs (siRNAs); but microRNAs (miRNAs) and long non-coding RNAs (lncRNAs) have been the most extensively characterized in cancer [38,39]. As their names suggest, miRNAs are small (18–20 nucleotides) while long non-coding RNAs are significantly longer (200–100,000 nucleotides). While these RNA species are divergent in their size and how they are post-transcriptionally processed, they share a common feature: a single miRNA or lncRNA is able to affect multiple genes/proteins [40,41]. Thus, deregulation of a single miRNA or lncRNA can influence many pathways and alter downstream processes such as apoptosis, proliferation, differentiation, etc. and act as either oncogene or tumor suppressor [42]. Many studies have described differential miRNA/lncRNA expression profiles between normal and cancerous human cells [43]. 

MicroRNAs repress protein production by binding to the 3′ untranslated region (3′-UTR) of their target messenger RNA (mRNA); this miRNA–mRNA duplex is both actively degraded and also prevents translation initiation [41]. While miRNAs are canonically repressive, lncRNA functions are more diverse. There are four archetypes of lncRNA function (decoy, activator, guide, or scaffold) which help dictate the interactions between transcription factors or chromatin modifier complexes; ultimately changing either the transcription of an mRNA or participating in post-transcriptional regulation of mRNA maturation processes [40].

Techniques that were originally developed to quantify mRNA have been adapted for quantification of miRNAs and lncRNAs. For detecting a broad range of non-coding RNAs, RNA sequencing (RNA-Seq) or microarrays are effective; however care should be taken with sample preparation and data analysis to ensure an accurate depiction of miRNAs/lncRNAs [44,45,46,47]. Secondary validation is necessary, often using real-time quantitative reverse transcription polymerase chain reaction (qRT-PCR) techniques to detect single non-coding RNAs of interest [48]. 

## 2. Risk Factors and Screening Strategies

Many factors influence cancer etiology including age, body mass index, physical activity, alcohol intake, smoking, environmental exposures, and family history [49,50,51,52,53]. Identifying and efficiently screening individuals who have the highest risk of developing cancer should improve the rate of early-stage diagnosis and translate to a direct survival benefit for the patient. Such a risk-stratified screening strategy could include epigenetic considerations as it is possible to identify and screen high-risk individuals with a pre-cancerous epigenotype. The success of these screening programs is dependent on tests that are: sensitive, low-cost, and relatively non-invasive. Here, minimally-invasive tests for epigenetic markers that could be used to screen high-risk individuals will be described.

### 2.1. Breast Cancer

In addition to regular mammograms, in several clinical experiments women at high-risk for developing breast cancer underwent ductal lavage, periareolar fine-needle aspiration, or a blood test. These high-risk women could be *BRCA1/2* mutation carriers, they could have a family history of the disease, or they may have had a previously identified hyperplasia or ductal carcinoma in situ [54,55].

When ductal lavage was performed on asymptomatic *BRCA1/2* mutation carriers, the cells within the lavage fluid of 8/19 women showed promoter hypermethylation of at least one of four genes (*BRCA1*, *BRCA2*, *ERα*, and *RARβ2*) that are often hypermethylated in breast cancer [56]. While none of the women subsequently developed breast cancer at the time of publication, 2/8 women with hypermethylation markers also presented ductal cell atypia in the lavage. In periareolar fine-needle aspirations from 86 high-risk women, there was actually no association between hypermethylation of three commonly hypermethylated breast cancer genes (*p16INK4a/ARF*, *BRCA1*, or *BRCA2*) and cellular atypia [57]. Strikingly, however, *p16INK4a* hypermethylation was significantly associated with hypermethylation of *BRCA1*, *BRCA2*, *ERα*, and *RARβ2* which is a candidate pre-cancerous hypermethylation profile. This suggests that promoter hypermethylation of candidate genes may be detectable before morphological changes and could be used to identify women who should be closely monitored for breast cancer. 

Blood samples are relatively easy to obtain, and while there are no approved serum- or blood-based markers for breast cancer screening, this is still an active area of research [58]. In a genome-wide methylation assessment of blood samples from high-risk women, 250 CpG sites were hypomethylated in individuals who went on to develop breast cancer [59]. Such a DNA methylation risk score could be used to intensify monitoring of certain women, once validated in independent cohorts. One study did use two independent cohorts to generate such a DNA methylation risk score for *BRCA1/2* mutation carriers. This signature was composed of 1722 CpG sites, and was relatively successful at predicting a breast cancer diagnosis within 12 years of the blood draw [60]. An early attempt to create a similar risk score with a serum-based model using 20 miRNAs was able to identify 31.7% of the women who developed breast cancer within 18 months of the blood draw [61].

### 2.2. Nasopharyngeal Carcinoma

Nasal endoscopy is the gold standard for detecting nasopharyngeal carcinoma; however this invasive procedure is not practical for population-level screening [62]. Nasopharyngeal swab collection is a low-cost and minimally-invasive alternative that has been used to screen high-risk individuals (family member of nasopharyngeal carcinoma patient) in a study by Yang et al. [63]. Promisingly, 66/96 patient swabs but 0/43 healthy family member swabs showed promoter hypermethylation in Ras association domain-containing protein 1 (*RASSF1*). This illustrates a low-cost way to monitor high-risk family members for nasopharyngeal carcinoma [64].

### 2.3. Lung Cancer

The current screening protocols for individuals at high-risk of developing lung cancer are based around serial computer tomography (CT) scans. Additional screening approaches are being investigated to supplement the use of CT imaging such as sputum analysis for DNA or atypical cells [65]. In a high-risk population with a cigarette smoking history of >30 pack years, hypermethylation of ≥3 genes (within a panel of *p16*, *MGMT*, *DAPK*, *RASSF1A*, *PAX5β*, and *GATA5*) in a sputum sample was associated with a 6.5-fold increase in the risk of developing lung cancer [66]. This suggests that further stratifying the high-risk smoking population could identify an extremely vulnerable population that would require intense monitoring. 

## 3. Diagnostic Biomarkers

### 3.1. Need for Liquid Biopsies

**Figure 1:**
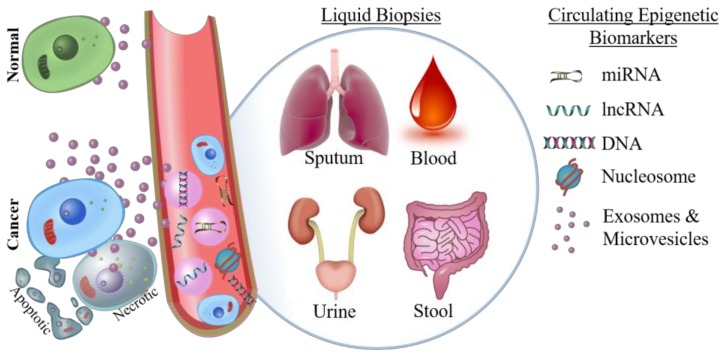
Types of epigenetic biomarkers that can be detected by liquid biopsy. Compared to normal cells, cancer cells shed a disproportionate amount of circulating free nucleic acids, microvesicle/exosome encapsulated nucleic acids, and nucleosomes; as well as shedding whole tumor cells into circulation. Epigenetic biomarkers can be detected in sputum (lung), urine (bladder and prostate), stool (colorectal), and blood (many cancer types). miRNA: microRNAs; lncRNA: long non-coding RNA.

Surgical resection or biopsy specimens are the gold standard for definitive cancer diagnosis, and this is unlikely to change in the near future. For example, core biopsy has a 93% sensitivity in the detection of breast cancer, and these samples can be used for subsequent immunohistochemical assessment to inform prognosis and therapy [67]. However, tissue biopsies are not always available; either due to anatomical location of the tumor making it difficult to safely biopsy, or because the risk of post-biopsy infection is unacceptable [68,69]. Therefore, liquid biopsies of blood, urine, sputum, or other bodily fluids are used as a less invasive way to detect biomarkers (Figure 1). 

An ideal biomarker for cancer detection should be easily and inexpensively measurable and should identify early-stage disease. It is essential that detection methods are sensitive to early-stage disease; as it is no coincidence that cancer types typically diagnosed at later stages (e.g., glioblastoma, pancreatic) also have the worst prognoses [70,71,72,73]. For epigenetic biomarkers to be suitable in liquid biopsy cancer detection, epigenetic dysregulation must occur early in carcinogenesis and the fluid epigenome should be reflective of the tumor epigenome [74]. 

### 3.2. Detecting Circulating Nucleotides and Nucleosomes

The presence of elevated cell-free DNA in serum of cancer patients was first observed 40 years ago [75], and is thought to be the result of ongoing release from apoptotic or necrotic cancer cells in the primary tumor [76]. Circulating free DNA (circ-DNA) in plasma, urine, and other bodily fluids is typically at a very low concentration and these double-stranded fragments are often of the length associated with nucleosomes (approximately 147 bp ± 20 bp linker DNA) with a portion of circ-DNA still complexed with histones [27,77,78].

Circulating miRNAs can be released passively by dying tissue or can be actively exported by cells. These miRNAs are remarkably stable in blood and seem to be protected from RNAses that should destroy free RNA [79,80,81,82]. This stability is because most miRNAs (approximately 90%) are bound to proteins such as Argonaute2, while others are encapsulated in vesicles such as exosomes while in circulation [83]. Circulating lncRNAs also seem to be detectable and stable in bodily fluids; however the mechanism of secretion and stability in the bloodstream is poorly understood and some studies could not reliably detect circulating lncRNAs [84]. 

Circulating histones are typically isolated with their associated DNA as a nucleosome. In cancer, circulating levels of nucleosomes are generally elevated, but the difference between healthy controls and patient nucleosome levels is not usually significant [85]. Determining the origins of circulating histones can be a difficult process. Only recently have methods emerged that can identify a nucleosome’s tissue-of-origin using “nucleosome footprinting” [86].

### 3.3. Colorectal Cancer

It was initially thought that the liquid biopsy epigenome would be an accurate proxy for the tumor epigenome; however concordance of tumor vs. serum markers is not observed in many studies [87,88]. Colorectal cancer has the highest degree of concordance between tumor and serum markers which has allowed for the rapid development of liquid biopsies to detect this cancer type [89]. Additionally, colorectal cancer has several qualities that make it a good model to develop novel diagnostic markers: there is a predictable pre-cancerous phase, there is low-compliance with current screening/detection programs, and current non-invasive markers are relatively insensitive [90].

There are five commercially available DNA methylation-based diagnostic tests for colorectal cancer: 3/5 of the commercial tests (Epi proColon, ColoVantage, and RealTime mS9) are designed to detect hypermethylated *SEPT9* in a blood sample. ColoSure detects hypermethylated vimentin in a stool sample, and Cologuard detects (among other markers) hypermethylated *NDRG4* and *BMP3* in stool [91,92,93,94,95,96,97,98]. While other biomarkers are being investigated in clinical trials, Epi proColon is the most clinically validated diagnostic epigenetic biomarker (Table 1). In a recent meta-analysis, serological *SEPT9* methylation was the superior methylation-based marker for colorectal cancer [99]. Tests like Epi proColon show great clinical potential because patients already prefer these non-invasive tests over colonoscopy/sigmoidoscopy for screening, and *SEPT9* methylation is superior at detecting early stage disease over other non-invasive tests like fecal occult blood testing [100,101,102]. Combinations of other hypermethylated genes as stool or blood biomarkers have not displayed the high sensitivity observed in the *SEPT9* methylation assays [89,103].

Other epigenetic biomarkers that could complement the methylation-based tests are being investigated. For example, serum levels of miR-21 were higher in colorectal cancer patients vs. individuals with benign polyps; however, it is not clear if this would detect early stage disease [104]. Due to limitations in histone isolation techniques, evidence for histone biomarkers lags behind DNA/RNA-based markers. Initial studies determined that H3K9me3 and H4K20me3 levels were significantly reduced in the plasma of colorectal cancer patients as compared to healthy controls [105,106]; but subsequent studies failed to confirm that H3K9me3 levels were different between patients and controls. Alternative histone marks, H3K27me3 and H4K20me3, only distinguished 49.2% of colorectal cancer patients [107].

### 3.4. Circulating Gene-Specific Promoter Hypermethylation

Unlike colorectal cancer, most other tumor types have low concordance between the methylation profile seen in tumor tissue vs. that observed in blood samples—this has limited the development of sensitive DNA methylation biomarkers. For example, several attempts have been made to use genes that are commonly hypermethylated in breast tumors (*GSTP1*, *RASSF1A*, *RARβ*, *APC*, and *DAPK*) to generate a blood-based breast cancer methylation signature [108]. Though at least one of these genes is methylated in 57–100% of tumors, methylation is detected in only 20–76% of patient serum samples [109,110,111]. One study used a different hypermethylated gene panel (*RASSF1A*, *ESR1*, *CDH1*, *TIMP3*, *SYK*) in the hopes of finding more concordant data; however, only 17% of patients in that study had simultaneous methylation of a gene in the tumor and in the blood [112].

Regardless of concordance with tumor tissue, serum-based multigene panels are more robust than single gene DNA methylation biomarkers. For instance, an eight gene promoter hypermethylation panel (*APC*, *BRCA1*, *BIN1*, *CST6*, *GSTP1*, *P16*, *P21*, and *TIMP3*) had a reported sensitivity of 90% for identifying breast cancer patients [74,113]. A similar approach using 17 gene promoters achieved a sensitivity of 91.2% and 90.8% specificity in distinguishing between pancreatic cancer patients and those with chronic pancreatitis [114,115].

Other than blood, several other fluids are candidates for liquid biopsy including: nasopharyngeal brushings, oral rinses, urine, vaginal fluid, and sputum. Nasopharyngeal brushings as well as oral rinses are a source of tumor-associated promoter hypermethylation to detect oral, pharyngeal, and nasopharyngeal carcinomas. DNA methylation markers (*RASSF1A*, *WIF1*, *DAPK1* and/or *RARβ2* + 20 additional CpGs) in these samples were predictive of early stage carcinoma when compared to healthy controls [63,116]. Urine could be a source of tumor DNA for detecting prostate and bladder cancer, though hypermethylation of glutathione *S*-transferase P1 (*GSTP1*) or other gene panels (e.g., *RASSF1A*, *APC*, *p14*) do not have sufficient sensitivity in detecting early stage disease [117,118]. Vaginal fluid could be used to distinguish endometrial or cervical cancer patients from healthy controls using a DNA methylation signature composed of 500 CpGs, which seemed to reflect methylation patterns seen in tumor biopsies [119]. Finally, sputum or bronchial lavage fluid can be used to aid in the diagnosis of lung cancer. Commercially available Epi proLung detects promoter methylation of short stature homeobox 2 (*SHOX2*) in bronchial lavage of lung cancer patients with a sensitivity of 78% and specificity of 96% [120].

### 3.5. Circulating Non-Coding RNAs

Similar to DNA hypermethylation markers, circulating non-coding RNA diagnostic markers are more robust when multiple non-coding RNAs are used, though some studies still utilize single markers. For example, elevated levels of miR-378 in serum can distinguish gastric cancer patients from healthy controls, while increased miR-141 identified prostate cancer [48,121]. Pancreatic cancer has been the focus of many miRNA biomarker studies, with more than 30 candidate miRNA blood biomarkers. Elevated levels of a single miRNA (miR-223) in plasma could distinguish patients with malignant intraductal papillary mucinous neoplasm (IPMN) from those with benign IPMN [122]. However, most of these potential miRNA markers are used within a panel, such as the combination of eight miRNAs (miR-6075, miR-4294, miR-6880-5p, miR-6799-5p, miR-125a-3p, miR-4530, miR-6836-3p, and miR-4476) which could achieve 80% sensitivity and 97.6% specificity in detecting pancreatic cancer [123,124]. Though detection of circulating lncRNAs is fraught with difficulties, plasma levels of lncRNA fragments HOXA distal transcript antisense RNA (HOTTIP-005) and RP11-567G11.1 were stably and significantly elevated in pancreatic cancer patients compared to healthy controls [125]. In gastric cancer patients, plasma levels of urothelial cancer associated 1 (UCA1/CUDR), long stress-induced non-coding transcript 5 (LSINCT-5), phosphatase and tensin homolog pseudogene 1 (PTENP1), and cytoskeleton regulator RNA (CYTOR/LINC00152) were higher than in healthy individuals [126,127]. However, it is unclear if circulating lncRNAs will ever be suitable blood-based biomarkers. 

### 3.6. Circulating Histone Modifications

Most studies have focused on identifying cancer-specific histone modifications using tumor tissue—due to the difficulty of isolating circulating histones. Unfortunately, this means that most data is going to be extrapolated from tumor biopsy to liquid biopsy without a thorough understanding of how concordant these samples are for histone modifications. For example, low H3K16Ac and H3K12Ac have been implicated as an early event in breast cancer progression, and proposed as circulating biomarkers [128,129]. Caution should be used when assuming tumor biopsy histone modifications are viable circulating biomarkers. 

Initial studies have focused on identifying histone modifications of circulating nucleosome isolated from cancer patients; though these modifications may not be effective at detecting early stage disease. For example, H3K9me3 and H4K20me3 have been detected on the circulating nucleosomes of multiple myeloma patients, but levels of these modifications were not compared the healthy controls and were not associated with disease stage [130]. In another study, the ratio of H3K9me3/nucleosome and H4K20me3/nucleosome was found to be higher in serum of breast and colorectal cancer patients compared to healthy individuals [131]. Similarly, an elevated ratio of H2AK119Ub/nucleosome H3K4me2/nucleosome was observed in pancreatic cancer patients [132]. 

Most histone variants have not been assessed as circulating biomarkers, so their utility is largely unknown; however, H2AZ has been assessed in a couple of studies. In the sera of colonoscopy patients, circulating levels of H2AZ did not distinguish between healthy controls and those diagnosed with colorectal cancer [107]. Circulating H2AZ levels (as part of an ELISA-based NuQ^®^ 5 nucleosome marker signature) were elevated in early stage pancreatic cancer patients [132]. 

Circulating histones and their cancer-specific modifications may have potential as biomarkers; however an association with early stage disease has not been established and the assays are more difficult and expensive than those used in isolating circulating nucleic acids.

## 4. Epigenetic Markers Informing Therapeutic Decision-Making

Epigenetic biomarkers may impact therapeutic decision-making through (Section 4.1) stratifying high-risk patients for intense treatment (Section 4.2) or predicting response to a specific treatment (Section 4.3). 

### 4.1. Epigenetic Prognostic Markers

Upon diagnosis, some cancer types are further classified based on immunohistochemical staining or other molecular markers. The resulting subtypes can be clinically relevant as in the case of breast cancer where the presence of estrogen receptor (ER), progesterone receptor (PR), and epidermal growth factor receptor (HER2) within the tumor directly impacts prognosis and treatment strategy [133]. Epigenetic profiling can complement these pre-existing prognostic biomarkers and further define the current subtypes; or there may be epigenetic markers that have prognostic relevance independent of our current subtyping strategies. 

Several epigenetic markers correlate with known prognostic factors in breast cancer. For example, high expression of lysine-specific histone demethylase 1, low levels of H3K9ac, H3K18ac, H4K12ac, H3K4me2, H4K20me2 and H4K3me2, and elevated amounts of circulating miR-21 and miR-10b are associated with the aggressive ER-subtype [83,128,134]. Epigenome-wide profiling studies of DNA methylation, histone modifications, miRNA expression, and lncRNA expression have generally found that unsupervised clustering of patients based on differential epigenome markers recapitulates the existing breast cancer subtypes [135,136]. 

Perhaps the best characterized epigenetic subtype is the CpG island methylator phenotype (CIMP) which was first identified in colorectal cancer [137]. CIMP+ tumors are identified based on promoter CpG island methylation of *CDKN2A* and *MLH1* genes, and these tumors tend to have a unique molecular and clinicopathological profile compared to CIMP lesions. While it is assumed that CIMP+ are generally more aggressive, some studies showed that once other important clinical factors were controlled for, CIMP+ status was not an independent predictor of poor prognosis [138]. 

There are plenty of other epigenetic markers that seem to be effective prognostic indicators such as high expression of lncRNAs HOTTIP-005 or RP11-567G11.1 and high serum miR196a predicting poor prognosis for pancreatic cancer [125,139]. While it is useful to define patients with poor prognoses and should encourage novel therapies for these underserved groups; here, the focus will be on describing epigenetic modifications that can more directly inform therapeutic strategies.

### 4.2. Anticipating the Benefit of Adjuvant Therapy

Epigenetic biomarkers could identify patients who would derive value from more intense or adjuvant therapies. In ER-/PR-/HER2- breast cancer patients, high miR-200b-3p and miR-190a along with low miR-512-5p levels in core biopsy was predictive of better response to neoadjuvant chemotherapy [140]. Similarly, in colorectal tumor samples, high expression of miR-16, miR-590-5p, and miR-153 predicted a complete response to neoadjuvant chemotherapy [141].

Retrospective analysis of patient cohorts which did not receive any adjuvant therapies can reveal patient subpopulations which would have benefitted from additional treatment. For example oral squamous cell carcinoma patients with a (*AJAP1*, *SHANK2*, *FOXA2*, *MT1A*, *ZNF570*, *HOXC4*, and *HOXB4*) methylation-based signature or stage I lung cancer patients with elevated H3K4me2 levels could have benefited from adjuvant chemotherapy [142,143]. Promoter methylation of *TNFRSF25*, *EDNRB*, *RASSF1A*, and *APC* could even be used to anticipate which bladder cancer patients require routine surveillance versus immediate radical treatment [144,145,146].

### 4.3. Epigenetic Biomarkers that Predict Response to Specific Therapies

Breast tumors that are immunohistochemically positive for ER expression are typically treated with the ER antagonist tamoxifen. It is unclear how methylation of the estrogen receptor 1 gene (*ESR1*) is connected with silencing of ER expression or response to tamoxifen. Unexpectedly, promoter hypermethylation of the *ER* gene is not generally predictive of decreased ER protein levels [147]; however, *ESR1* methylation in circulating DNA actually does correlate with ER protein in the tumor [148]. It was hypothesized that *ER* silencing via *ESR1* hypermethylation could indicate resistance to tamoxifen; but unexpectedly, *ESR1* methylation was predictive of longer survival in tamoxifen-treated patients [149]. 

Cytotoxic chemotherapies like taxanes preferentially affect cancer cells based on their rapid proliferation and cell cycle checkpoint dysfunction. Checkpoint with forkhead and ring finger domains (CHFR) controls cell cycle progression at the G2/M checkpoint and can initiate mitotic arrest; thus downregulation of CHFR encourages uncontrolled cellular division [150,151]. Hypermethylation of *CHFR* is observed in breast, bladder, colorectal, gastric, nasopharyngeal, lung, esophageal, cervical, hepatocellular, oral squamous, head and neck, and endometrial cancer [152]; and is a potential marker of taxane sensitivity for many cancer types. In vitro evidence using nasopharyngeal carcinoma, colorectal cancer, esophageal, endometrial cancer, and gastric cancer cell lines suggests that *CHFR* hypermethylation contributes to docetaxel and paclitaxel sensitivity [153,154,155,156,157]. However, subsequent clinical studies have not shown a clear influence of *CHFR* methylation on taxane sensitivity. In advanced gastric cancer patients, *CHFR* methylation was not predictive of response to docetaxel or paclitaxel [158]. Clearly, randomized prospective clinical trials are necessary to confirm the clinical validity of *CHFR* methylation as a marker of taxane sensitivity [159]. 

Expression of the excision repair enzyme *O*-6-methylguanine–DNA methyltransferase (MGMT) has previously been associated with clinical resistance to alkylating agents [160]. This suggested that epigenetic silencing of *MGMT* may cause sensitivity to those same drugs [161]. This seems to generally be the case for glioblastoma patients as *MGMT* promoter hypermethylation is an independent favorable prognostic factor [162,163]. Patients with promoter methylation survived longer after temozolomide plus radiotherapy treatment compared to patients with unmethylated *MGMT*. Similarly, *MGMT* hypermethylation was observed in gliomas from patients who had survived longer after treatment with the alkylating agent carmustine [160]. However, not all trials have shown predictive value of *MGMT* promoter methylation [164]. 

## 5. Epigenetic Biomarkers to Monitor Patient Responses

Liquid biopsies could be used to monitor the real-time dynamics of a patient’s response to therapy or to monitor survivors for potential recurrence. Early response to therapy is often indicative of an overall better prognosis, so early response biomarkers would be a valuable prognostic tool and could also be used to make timely adjustments to treatment regimens [165]. For example, in patients with advanced lung cancer, a significant decrease in circulating nucleosome levels was indicative of a favorable response to the first chemotherapy cycle [166].

Epigenetic markers could be applied in two ways when monitoring for relapse: patients could be stratified based on risk of recurrence and routine monitoring could be intensified, or unique epigenetic markers could identify patients who are currently experiencing relapse. In urothelial carcinomas, hypermethylated insulin-like growth factor-binding protein 3 (*IGFBP3*) and apoptotic protease activating factor 1 (*APAF-1*) was indicative of a high risk of recurrence and identified patients who should be monitored closely; however, this was not a liquid biopsy and close monitoring could not be aided with epigenetic biomarkers [167]. Peritoneal fluid is relatively accessible and sentinel lymph nodes are occasionally dissected in gastrointestinal cancer patients, so several groups have used these samples to assess patients for recurrence. In the peritoneal fluid or sentinel lymph nodes of gastric cancer patients, hypermethylation of a 6-gene signature predicted an increased risk of recurrence or metastasis [168,169]. Blood samples are very accessible, and global hypermethylation was associated was increased relapse potential in acute lymphoblastic leukemia patients; however, global hypomethylation (*LINE-1*) was associated with relapse risk in oropharyngeal squamous cell carcinoma [170,171]. There are several markers that show promise for identifying patients who are experiencing relapse, such as three miRNAs that were upregulated and 8 miRNAs that were downregulated in the serum of recurrent breast cancer patients [172].

## 6. Future Directions

The detection and assessment of circulating nucleic acids is still in the developmental stage and will require standardization of methodologies before true clinical utility is achieved. Conclusive evidence for candidate biomarkers is often missing because meta-analyses are limited by the wide range of methodologies used between research groups. For example, there is not even a consensus on whether serum or plasma is a more appropriate sample type for DNA or lncRNA biomarkers; and there no clear evidence for the significance (if any) of contaminating leukocyte DNA [173]. So far, several recommendations have been made: miRNA biomarkers should be assayed from serum samples [48,83,174], and DNA methylation biomarker panels should not explicitly include leukocyte differentially methylated regions [175,176].

Finally, even though DNA methylation biomarkers have advanced into the clinic (e.g., Epi proColon); unbiased approaches using newer techniques should identify additional DNA methylation biomarkers. Most candidate genes in methylation biomarker panels were chose a priori based on the gene product’s function, and while these candidate gene approaches have led to the development of successful biomarkers, care should be taken in their interpretation. Due to the volume of differentially methylated sites and lack of established *p*-value correction protocols, one study showed that 30–50% of randomly selected methylation sites could be significantly associated with clinical factors [177]. We should not only re-assess our current biomarker panels, but also use unbiased approaches with stringent statistics and large patient cohorts. Excitingly, DNA methylation biomarkers can finally be directly functionally validated with the creation of a clustered regularly interspaced short palindromic repeats (CRISPR) method which uses site-specific targeting with DNA methyltransferase 3 (DNMT3A) or methylcytosine dioxygenase 1 (TET1) to add/subtract a methyl group [178,179]. These novel techniques should finally resolve which methylation sites play a vital role in cancer progression and could reveal novel biomarkers. 

## Figures and Tables

**Table 1:** Clinical trials using diagnostic, prognostic, or predictive biomarkers based on DNA methylation.

Clinical Trial ID	Marker	Test	Sample	Neoplasm	Biomarker Type	Result/Status
NCT01329718	Hypermethylated Septin-9 (*SEPT9*)	Epi proColon	Blood	Colorectal cancer	Diagnostic	Completed
NCT03218423				Colorectal cancer	Longitudinal (Diagnostic)	Recruiting
NCT00696345				Colorectal cancer	Diagnostic	Completed
NCT02198092				Hereditary colorectal cancer	Diagnostic	Recruiting
NCT03311152				Hepatocellular carcinoma	Diagnostic	Recruiting
NCT02540850	Hypermethylated *SEPT9*		Blood	Colorectal cancer	Diagnostic	Completed
NCT00855348				Colorectal cancer	Diagnostic	Completed
NCT02419716	Hypermethylated NDRG family member 4 (*NDRG4*), bone morphogenetic protein 3 (*BMP3*)	ColoGuard	Stool	Colorectal cancer	Diagnostic	Active/Not Recruiting
NCT02715141						Recruiting
NCT01793207	7 Cpgs		Blood	Colorectal cancer	Diagnostic	Completed
NCT03146520	Hypermethylated syndecan 3 (*SDC2*)	EarlyTect	Stool	Colorectal cancer	Diagnostic	Enrolling by invitation
NCT02159339	Hypermethylated cyclin dependent kinase inhibitor 2A (*p16*)		Tumor	Gastric cancer	Prognostic- Metastasis	Completed
NCT00835341			Biopsy	Oral cancer	Diagnostic	Completed
NCT01695018			Mucosal Biopsy	Oral cancer	Diagnostic	Completed
NCT01774266	Hypermethylated TP53-dependent G2 arrest mediator homolog (*REPRIMO*)		Serum	Gastric cancer	Diagnostic	Recruiting
NCT01715233	Hypermethylated checkpoint with forkhead and ring finger domains (*CHFR*)		Biopsy	Esophageal, gastroesophageal, gastric cancer	Predictive-Taxane Response	Recruiting
NCT01372202			Biopsy	Esophageal cancer		Active/Not Recruiting
NCT03217097	Hypermethylated O-6-methylguanine-DNA methyltransferase (*MGMT*)		Biopsy	Neuroendocrine tumors	Predictive-Oxaliplatin/Alkylating Agent Response	Not Yet Recruiting
NCT02700464	15 CpGs	EpiCheck	Urine	Bladder eurothelial cell carcinoma	Recurrence	Recruiting
NCT02647112						Recruiting
NCT02688491	5 CpGs		Surgical Specimen	Clear cell renal carcinoma (Stage III)	Predictive-Sunitinib Response	Not Yet Recruiting
